# Supplementary material for: BCG vaccination-induced acquired control of mycobacterial growth differs from growth control preexisting to BCG vaccination
Source: Nat Commun. 2024 Jan 2;15:114. doi: 10.1038/s41467-023-44252-5 (PMC10761850; doi:10.1038/s41467-023-44252-5)
Supplement: Supplementary file 1 — Supplementary Information [file 41467_2023_44252_MOESM1_ESM.pdf]

Supplementary table 1: Antibody panel information Indian cohort

Marker	Fluorochrome	Clone	Company	Catalog #	Dilution
**Live/dead staining (RT):**					
L/D	Fixable Blue	-	Thermo Fisher	L23105	1000
**Chemokine receptor staining (37°C):**					
CCR4	PE-Cy7	L291H4	Biolegend	359410	50
CCR6	BV605	G034E3	Biolegend	353420	50
CCR7	BV421	G043H7	Biolegend	353208	50
CXCR3	BUV661	1C6/CXCR3	BD Biosciences	741649	50
CCR2	BV785	K036C2	Biolegend	357234	200
CX3CR1	PerCP-Cy5.5	2A9-1	Biolegend	341614	100
**Surface staining (4°C):**					
CD3	BV510	UCHT1	Biolegend	300448	50
CD4	CF568	EDU-2	Biotium	BNC680345	400
CD8	BUV805	G42-8	BD Biosciences	749032	400
CD45RA	BUV395	5H9	BD Biosciences	740315	400
CD27	Pacific Blue	O323	Biolegend	302822	100
CD28	BUV563	L293	BD Biosciences	748476	50
CD95	PE-Cy5	DX2	Biolegend	305610	400
CD62L	APC-Fire750	DREG-56	Biolegend	304846	100
CD25	PE-Fire640	M-A251	Biolegend	356148	50
CD300c	BV650	TX45	BD Biosciences	743658	50
CD56	BV750	5.1H11	Biolegend	362556	100
CD16	BUV496	B73.1	BD Biosciences	741207	50
CD14	Spark Blue 550	63D3	Biolegend	367148	400

CD85d	PE-Dazzle 594	42D1	Biolegend	338710	100
HLA-DR	PE-Fire810	L243	Biolegend	307683	100
CD38	APC-Fire810	HIT2	Biolegend	303550	100
CD163	APC	GHI/61	Biolegend	333610	100
CD19	Spark NIR 685	HIB19	Biolegend	302270	100
CD20	Pacific Orange	HI47	Life Technologies	MHCD2030	50
IgD	BV480	IA6-2	BD Biosciences	566187	100

Supplementary table 2: Antibody panel information 300BCG Dutch cohort

Marker	Fluorochrome	Clone	company	catalogue #	dilution
**Panel 1**					
CD16	FITC	3G8	Coulter	6604894	50
HLA-DR	PE	immu-357	Coulter	IM1639	10
CD14	ECD	RMO52	Coulter	B92391	100
CD4	PE-Cy5.5	13B8.2	Coulter	B16491	200
CD25	PC7	M-A251	BD Biosciences	560920	50
CD56	APC	N901	Coulter	IM2474	50
CD8	APC-AF700	B9.11	Coulter	B49181	400
CD19	APC-AF750	J3-119	Coulter	A94681	50
CD3	Pacific Blue	UCHT1	Coulter	B49204	50
CD45	Krome Orange	J33	Coulter	B36294	50
**panel 2**					
CD45RA	FITC	ALB11	Coulter	A07786	50
CD3	PE	UCHT1	Coulter	A07747	50
CD45RO	ECD	UCHL1	Coulter	B49192	50
CD27	PE-Cy5.5	1A4CD27	Coulter	B21444	100
CD25	PC7	M-A251	BD Biosciences	560920	50
CD56	APC	N901	Coulter	IM2474	50
CD127	APC-AF700	R34.34	Coulter	A71116	50
CD8	APC-AF750	B9.11	Coulter	A94683	400
CD4	Pacific Blue	13B8.2	Coulter	B49197	100
CD45	Krome Orange	J33	Coulter	B36294	50
**panel 3**					
IgD	FITC	IA6-2	Coulter	B30652	50
IgM	PE	SA-DA4	Coulter	B30657	50
CD3	ECD	UCHT1	Coulter	A07748	50
CD27	PE-Cy5.5	1A4CD27	Coulter	B21444	100
CD38	PC7	LS198-4-3	Coulter	B49198	25
CD24	APC	ALB9	Coulter	A87785	25
CD5	APC-AF700	BL1a	Coulter	A78835	50
CD19	APC-AF750	J3-119	Coulter	A94681	50
CD20	Pacific Blue	B9E9	Coulter	B49208	50
CD45	Krome Orange	J33	Coulter	B36294	50
**panel 4**					
KI67	AF488	B56	BD Biosciences	558616	50
HLA-DR	PE	immu-357	Coulter	IM1639	10
CD45RA	ECD	2H4LDH11LD89	Coulter	B49193	50
CD4	PE-Cy5.5	13B8.2	Coulter	B16491	200
CD25	PC7	M-A251	BD Biosciences	560920	50
Helios	AF647	22F6	BD Biosciences	563951	40

CD127	APC-AF700	R34.34	Coulter	A71116	50
CD8	APC-AF750	B9.11	Coulter	A94683	400
FoxP3	eFluor450	PCH101	eBioscience	48-4776-42	50
CD45	Krome Orange	J33	Coulter	B36294	50
**panel 5**					
CD16	FITC	3G8	Coulter	6604894	25
CD10	PE	HI10A	BioLegend	982206	25
CD11b	PE-Dazzle	ICRF44	BioLegend	301348	50
CD14	PE-Cy5.5	M5E2	BioLegend	301864	50
CD62L	PC7	DREG-56	BioLegend	304822	50
CD274	APC	MIH1	ThermoFisher	17-5983-42	25
CD66b	AF700	G10F5	BioLegend	305114	50
CD15	BV421	W6D3	BioLegend	323040	50
CD45	Krome Orange	J33	Coulter	B36294	50

Supplementary figure 1. Schematic overview of study design and analysis

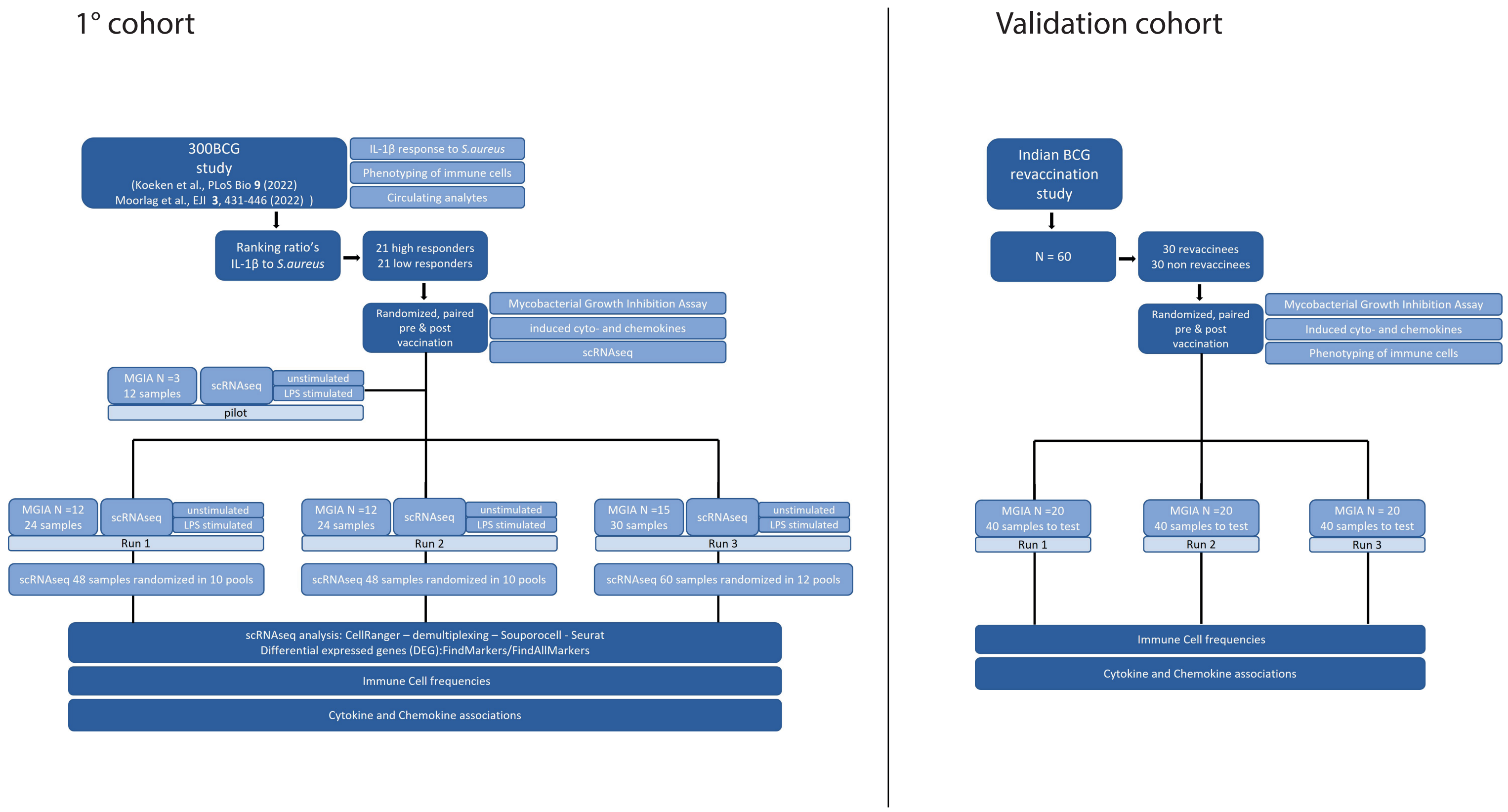

Supplementary Figure 2

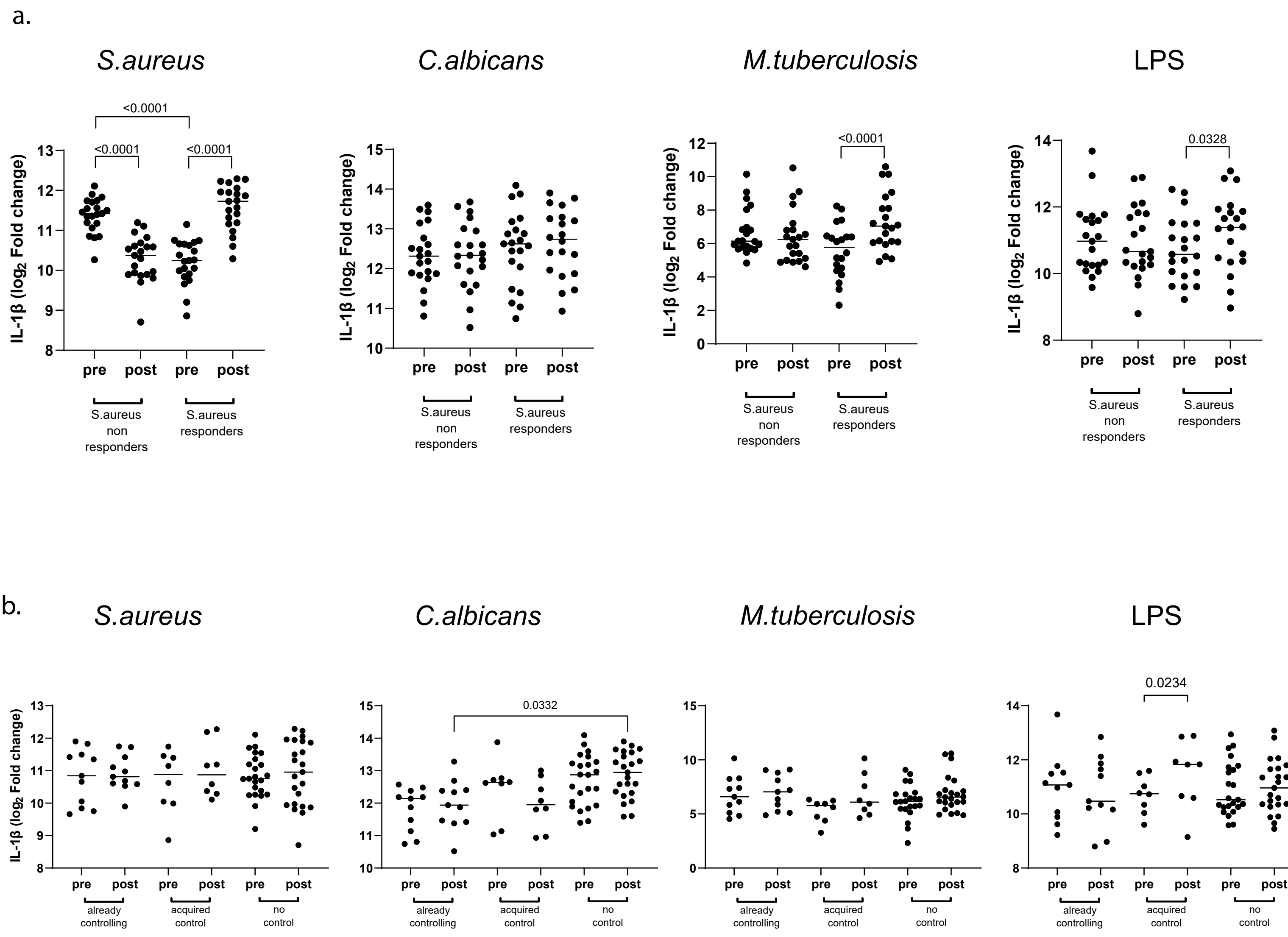

Supplementary Fig.2: Antigen stimulation responses for 300BCG cohort. PBMCs were stimulated for 24 hours with heat-killed *S.aureus*, *C.albicans*, *M.tuberculosis* or LPS. IL-1β was measured in culture supernatants. (a) Scatter plots of the responses pre and post BCG vaccination divided into the two groups based on *S.aureus* responses. IL-1β concentrations are expressed as log2 fold change over unstimulated control, (b) Scatter plots of the IL-1β responses as plotted in (a) but according to the functional groups for BCG outgrowth control. Statistical analysis was performed within groups (Wilcoxon test, two-sided) and between groups (Mann-Whitney, two-sided) with significant p-values depicted in the graphs.

Supplementary Figure 3

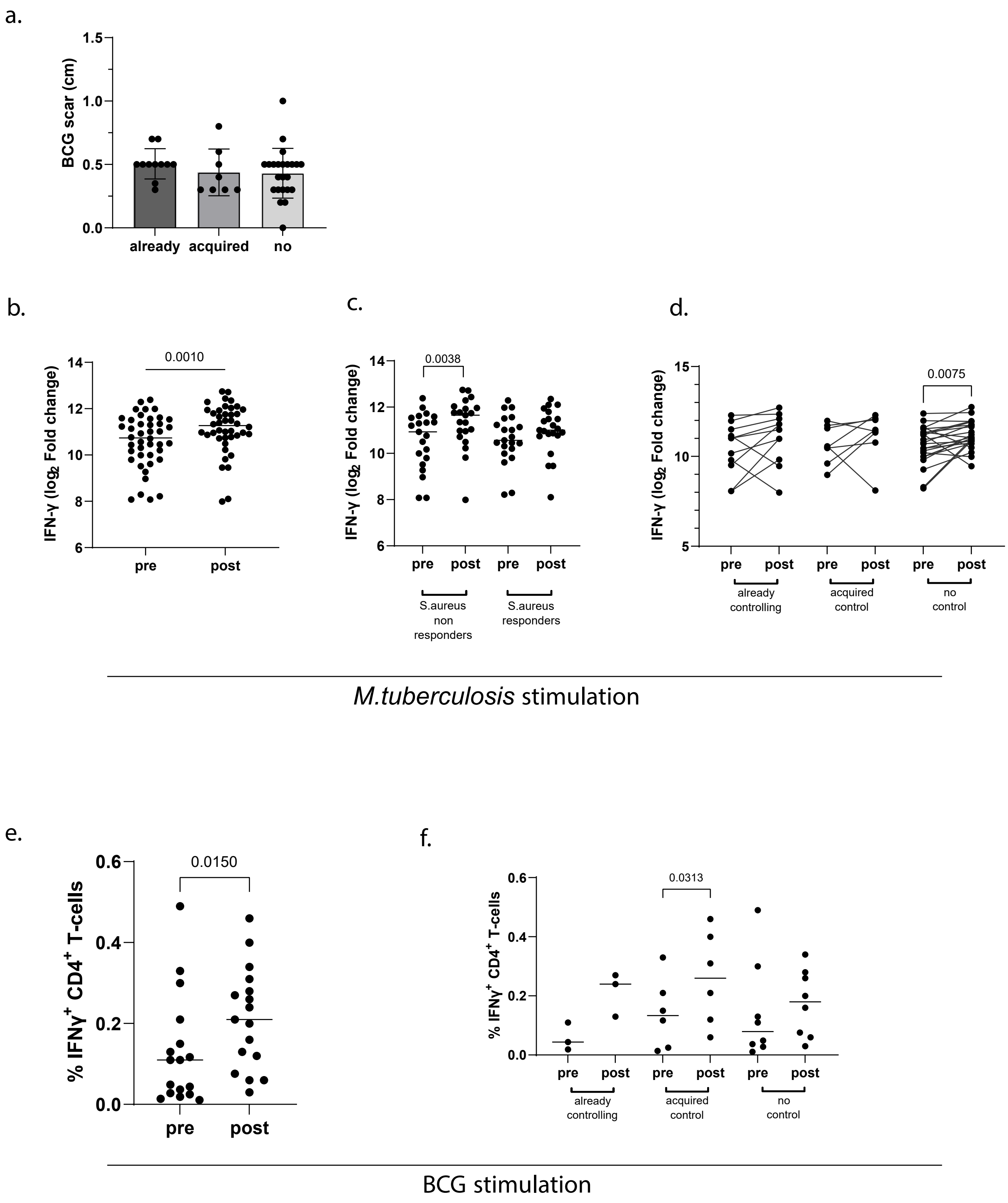

Supplementary Fig.3: BCG vaccine take for 300BCG and Indian revaccination cohorts. (a) BCG scar information (cm) for the primo BCG vaccination in the 300BCG cohort is shown for the three functional groups. **(b)** PBMCs of 300BCG individuals were cultured for 7 days with heat-killed *M.tuberculosis* and IFN-γ was measured in the culture supernatants and is shown as log2 fold change over the unstimulated control. These *M.tuberculosis* induced IFN-γ responses are also shown in relation to the *S.aureus* responder classification **(c)** and the functional groups **(d)**. For the Indian revaccination cohort whole blood samples pre and 8-12 weeks post BCG-revaccination were incubated with BCG for 12 hours and IFN-γ production by $CD4^+$ T-cells was determined by flow cytometry **(e)** and plotted for the three functional groups **(f).** Within group comparisons were tested using Wilcoxon matched-pairs test (two-sided) to determine the p values.

Supplementary Figure 4

a.

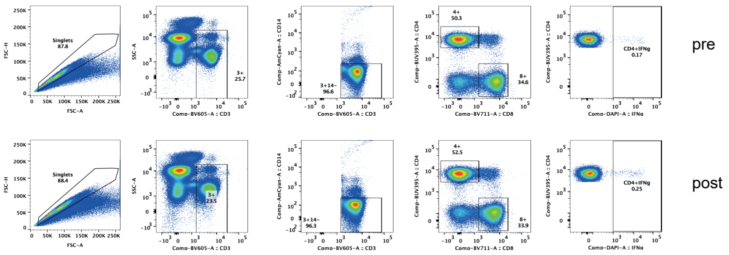

b.

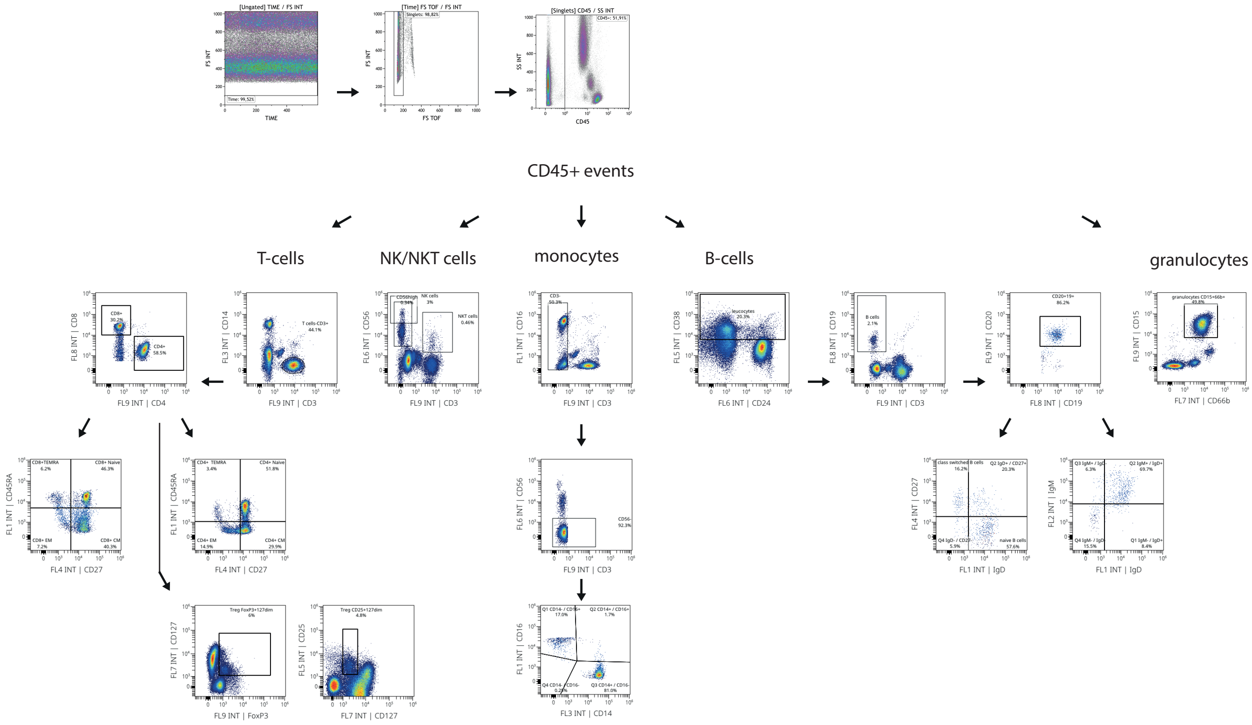

c.

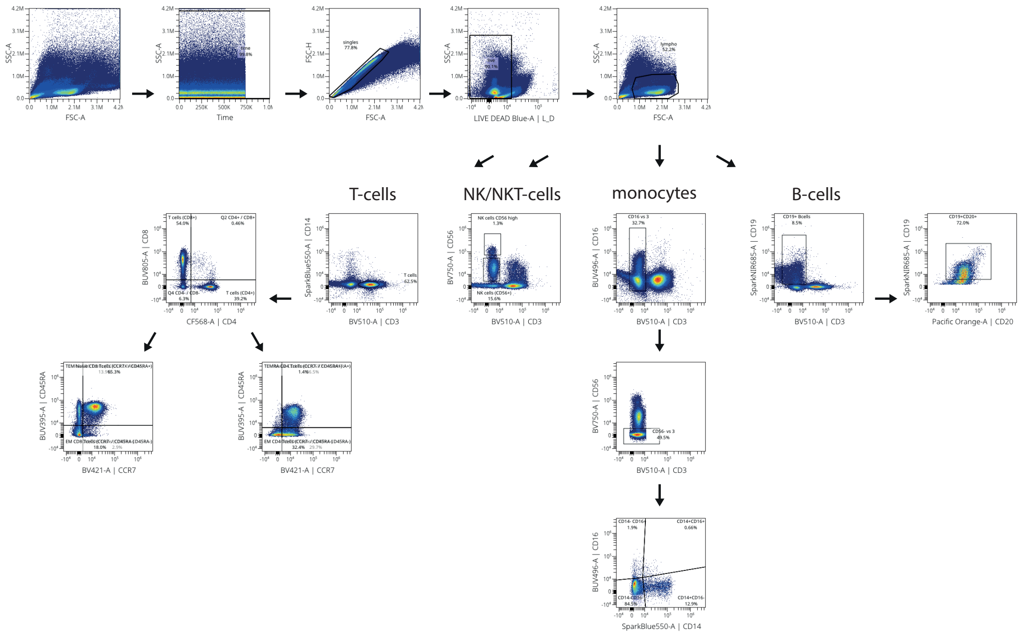

Supplementary Fig.4: Gating strategies flow cytometry analysis. (a) Gating strategy for the vaccine take responses of the Indian validation cohort. **(b)** Gating strategy for cellular subsets of the Dutch 300BCG cohort. **(c)** Gating strategy for the cellular subsets of the Indian BCG-revaccination cohort.

Supplementary Figure 5

a.

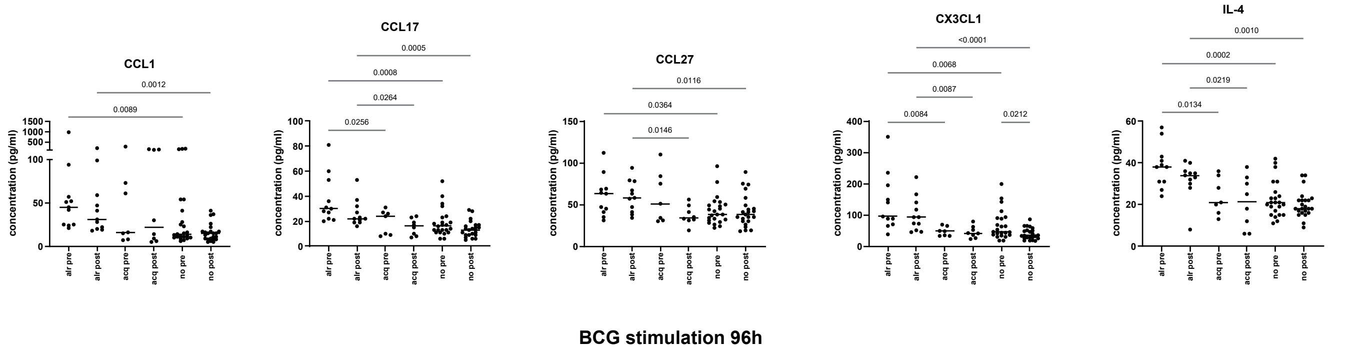

b.

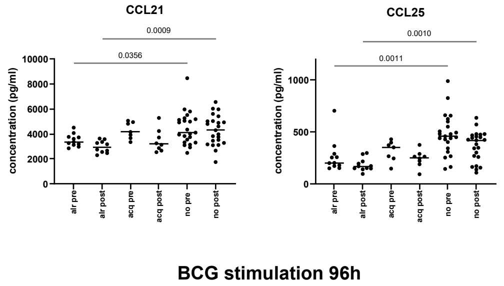

Supplementary Fig.5: : Representative of the chemo- and cytokine responses. Examples of the chemo- and cytokine levels are shown as univariate scatterplots for the 300BCG cohort, analytes with relative high concentrations in already controllers pre vaccination **(a)** and relatively low concentrations in BCG controllers post vaccination **(b)**. Statistical analysis was performed by a two-sided Kruskall-Wallis test with uncorrected Dunn's test.

Supplementary Figure 6

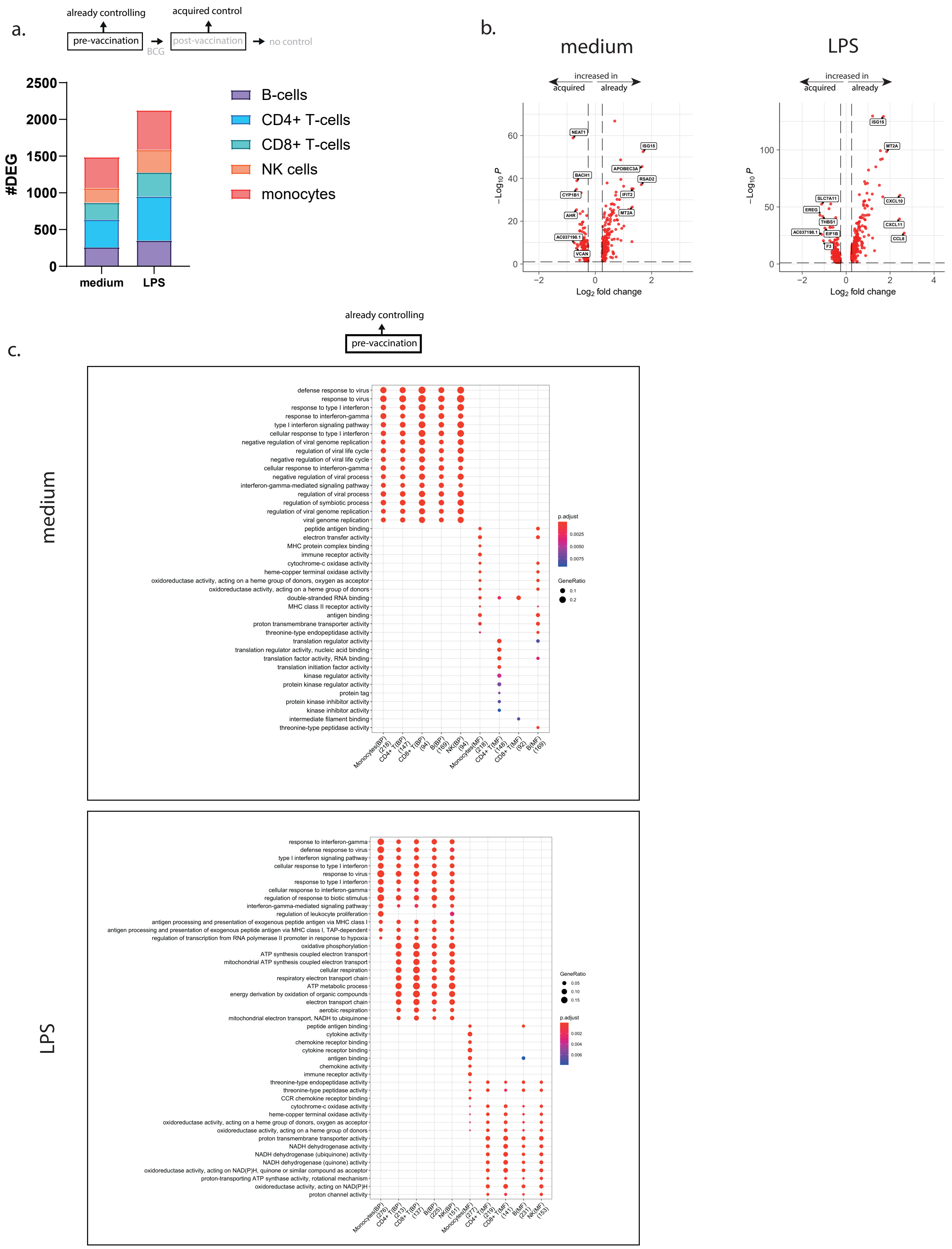

Supplementary Fig.6: Prior to vaccination the transcriptome profile of already controllers is intrinsically different from acquired controllers. PBMCs were stimulated with medium or LPS for 4 hours and processed for single cell RNA seq analysis. **(a)** Stacked bars represent the differentially expressed gene number after deconvolution of cell subsets, between already controllers vs. that acquired controllers at the BCG vaccination time point, colors indicate the respective subsets. **(b)** Scatter-volcano plots showing the significance (-log$_{10}$*P*) versus magnitude of change (Log$_2$ fold change) of the differentially expressed genes in the monocytes for both unstimulated (left plot) vs. LPS stimulated samples (right plot), statistical testing performed by Wilcoxon Rank Sum test (two-sided) and genes were considered significant when expressed in at least 10% of the cells and an adjusted p-value <0.05 after Benjamini-Hochberg correction. Genes with a log fold change <0.25 are increased in individuals that will acquire BCG control upon vaccination, genes with a log fold change >0.25 are increased in already controllers. **(c)** Pathway analysis showing the biological processes (BP) and molecular functions (MF) for the different cell subsets for the unstimulated and the LPS stimulated samples, related to the differentially expressed and upregulated genes in **(b)** for the already controllers. Dot colours represent adjusted p-values and dot sizes the gene ratios. For every subset the number of genes involved is shown between brackets.

Supplementary Figure 7

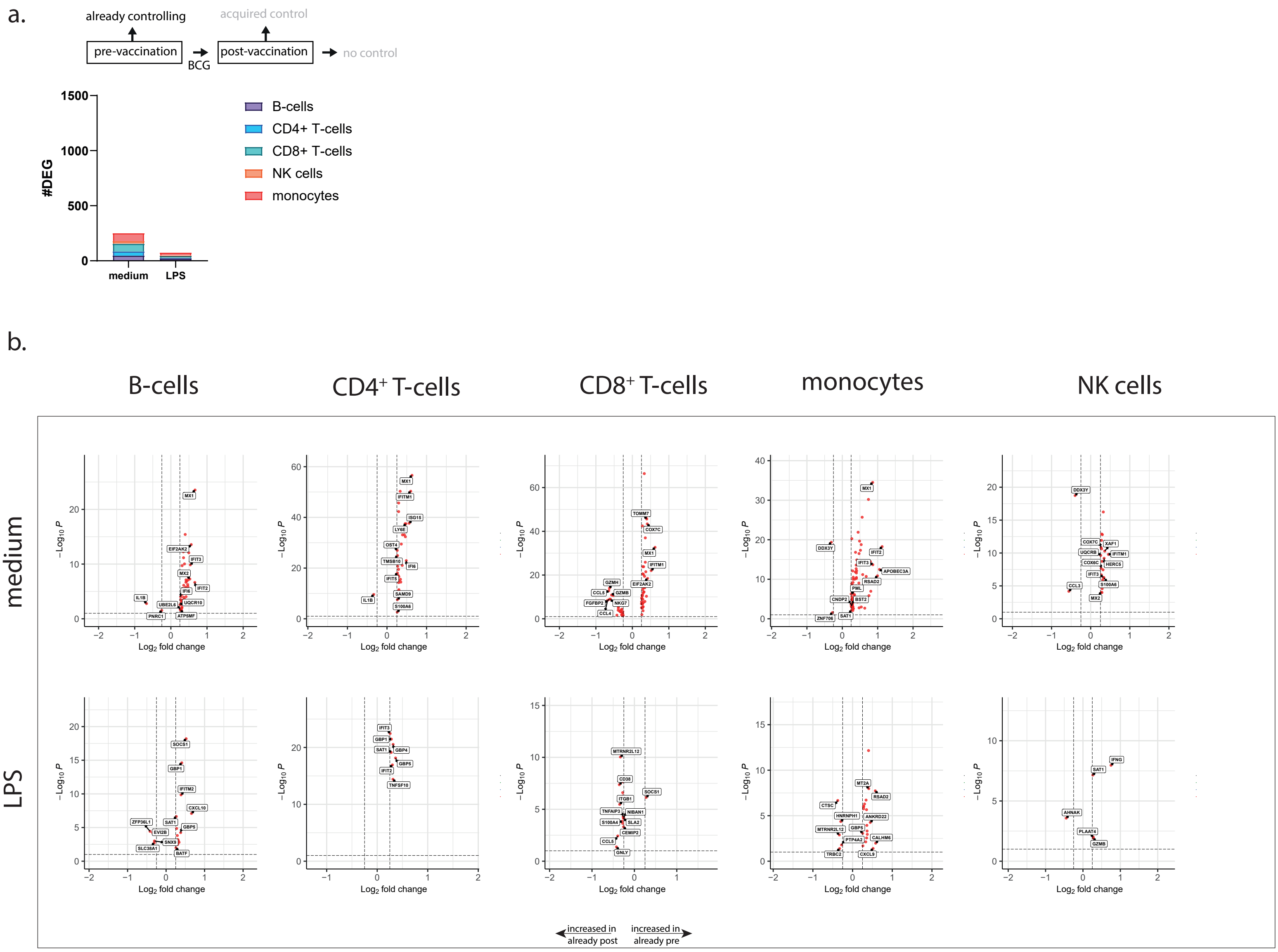

Supplementary Fig.7: BCG vaccination induced minimal changes in gene expression in already controllers. PBMCs were stimulated with medium or LPS for 4 hours and processed for single cell RNA seq analysis. **(a)** Stacked bars represent the differentially expressed gene number after deconvolution of cell subsets, between already controllers vs. that acquired controllers at the BCG vaccination time point, colors indicate the respective subsets. **(b)** Scatter-volcano plots showing the significance (-$\log_{10}P$) versus magnitude of change (Log_2 fold change) of the differentially expressed genes for both the unstimulated (upper panel) vs. the LPS stimulated samples (lower panel) for the B cells, $CD4^+$ and $CD8^+$ T cells, monocytes and NK cells. Statistical testing was performed by Wilcoxon Rank Sum test (two-sided) and genes were considered significant when expressed in at least 10% of the cells and an adjusted p-value <0.05 after Benjamini-Hochberg correction. Genes with a log fold change <0.25 are increased in already controlling individuals after BCG vaccination, genes with a log fold change >0.25 are increased prior to BCG vaccination.

Supplementary Figure 8

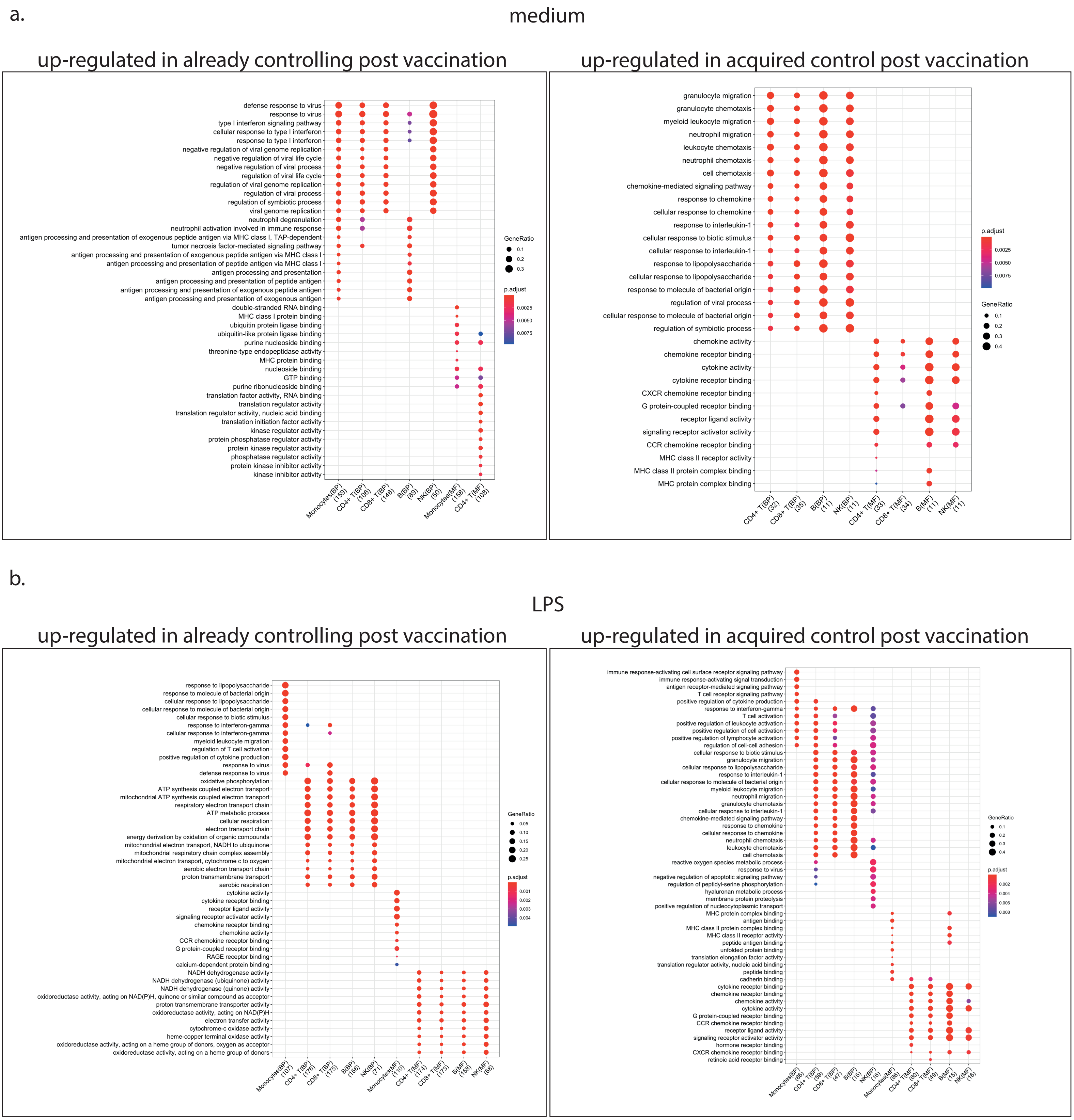

Supplementary Fig.8: Pathways differ between already controllers and acquired controllers after BCG vaccination. PBMCs were stimulated with medium or LPS for 4 hours and prepared for single cell RNA seq analysis. Pathway analysis showing the biological processes (BP) and molecular functions (MF) for the different cell subsets for unstimulated samples **(a)** and LPS stimulated samples **(b)**, after BCG vaccination for both the already controllers as the acquired controllers. The dot colour representing the adjusted p-values and the dot size the gene ratio. For every subset the number of genes involved is shown between brackets and for the enrichment test, significant gene sets were subjected to enrichGO and enrichKEGG function (pAdjustMethod = “BH”, qvalueCutoff = 0.05) and pathways with BH correction (FDR) < 0.05 were considered significant.

Supplementary Figure 9

a.

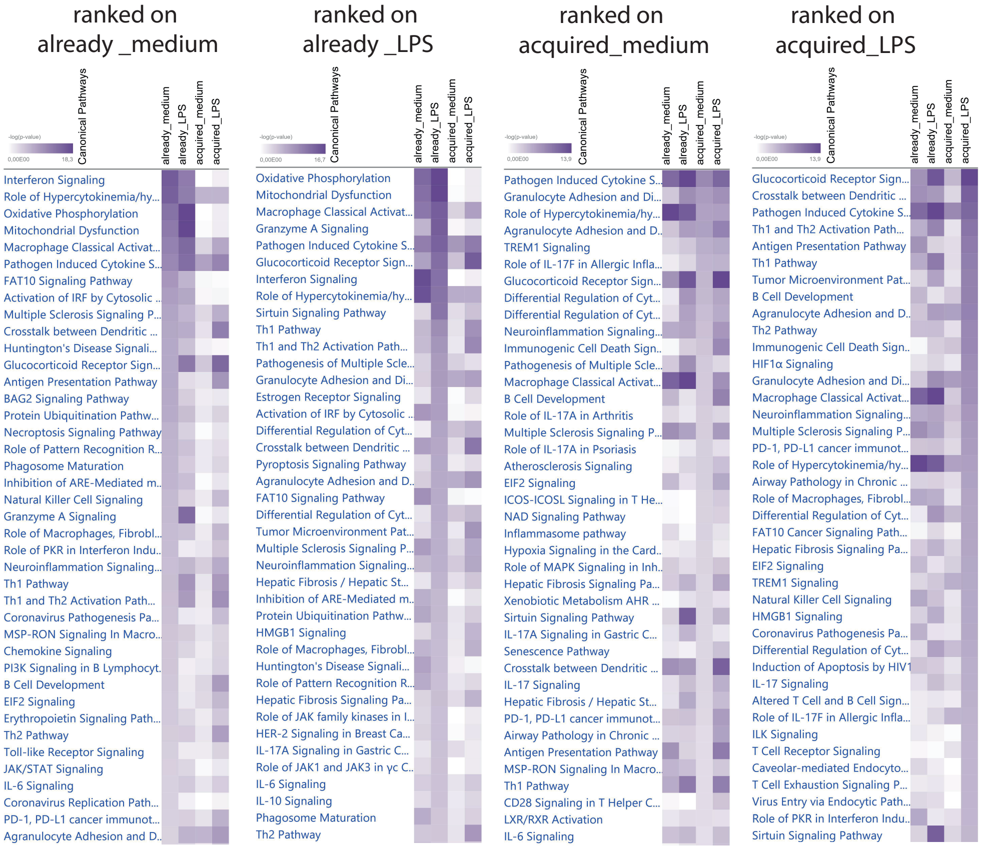

b.

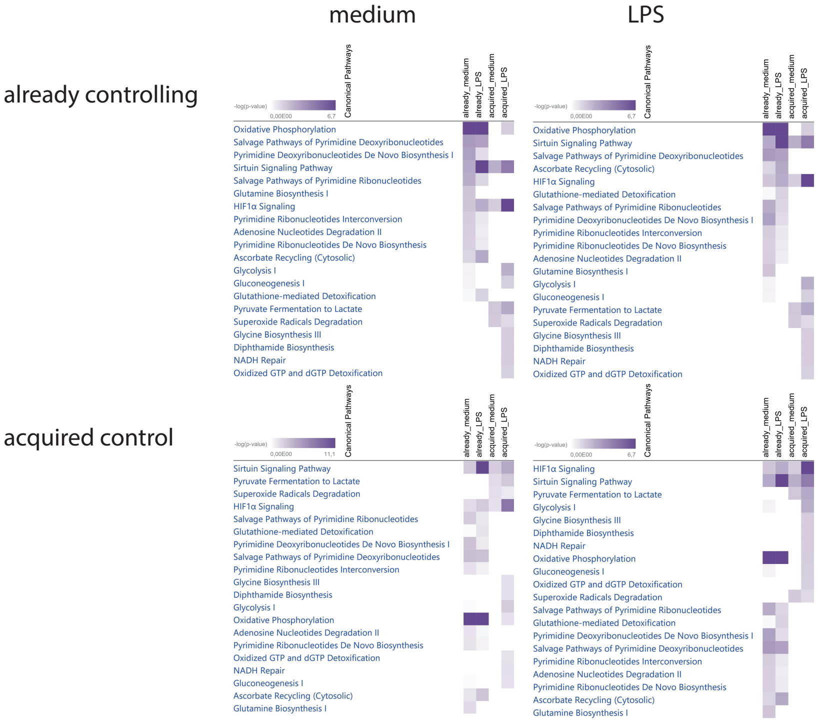

Supplementary Fig.9: Comparison analysis for already controllers and the acquired controllers. Heatmaps generated in Ingenuity Pathway Analysis after core analysis and a comparison analysis. **(a)** showing the top 40 most enriched canonical pathways ranked for the already controllers prior to and the acquired controllers after BCG vaccination with or without stimulation. **(b)** Focussing on the metabolic processes ranked on the unstimulated or LPS stimulation in the already controllers (top) or the acquired controllers (bottom). All based on the differentially expressed genes involved in the BCG outgrowth control, with the purple shading representing the -log(p-value).
