# Supplementary material for: BCG vaccination-induced acquired control of mycobacterial growth differs from growth control preexisting to BCG vaccination
Source: Nat Commun. 2024 Jan 2;15:114. doi: 10.1038/s41467-023-44252-5 (PMC10761850; doi:10.1038/s41467-023-44252-5)
Supplement: Supplementary file 3 — Reporting Summary [file 41467_2023_44252_MOESM3_ESM.pdf]

nature portfolio

Corresponding author(s): SA Joosten

Last updated by author(s): 27-10-2023

Reporting Summary

Nature Portfolio wishes to improve the reproducibility of the work that we publish. This form provides structure for consistency and transparency in reporting. For further information on Nature Portfolio policies, see our Editorial Policies and the Editorial Policy Checklist.

Please do not complete any field with "not applicable" or n/a. Refer to the help text for what text to use if an item is not relevant to your study.
For final submission: please carefully check your responses for accuracy; you will not be able to make changes later.

Statistics

For all statistical analyses, confirm that the following items are present in the figure legend, table legend, main text, or Methods section.

n/a	Confirmed	
☐	☒	The exact sample size (*n*) for each experimental group/condition, given as a discrete number and unit of measurement
☒	☐	A statement on whether measurements were taken from distinct samples or whether the same sample was measured repeatedly
☐	☒	The statistical test(s) used AND whether they are one- or two-sided<br>*Only common tests should be described solely by name; describe more complex techniques in the Methods section.*
☐	☒	A description of all covariates tested
☐	☒	A description of any assumptions or corrections, such as tests of normality and adjustment for multiple comparisons
☐	☒	A full description of the statistical parameters including central tendency (e.g. means) or other basic estimates (e.g. regression coefficient) AND variation (e.g. standard deviation) or associated estimates of uncertainty (e.g. confidence intervals)
☐	☒	For null hypothesis testing, the test statistic (e.g. *F*, *t*, *r*) with confidence intervals, effect sizes, degrees of freedom and *P* value noted<br>*Give P values as exact values whenever suitable.*
☒	☐	For Bayesian analysis, information on the choice of priors and Markov chain Monte Carlo settings
☒	☐	For hierarchical and complex designs, identification of the appropriate level for tests and full reporting of outcomes
☒	☐	Estimates of effect sizes (e.g. Cohen's *d*, Pearson's *r*), indicating how they were calculated

Our web collection on statistics for biologists contains articles on many of the points above.

Software and code

Policy information about availability of computer code

Data collection: Bio -plex manager v6.1 (Bio-Rad laboratories); Olink® NPX Signature Software (Olink); Kaluza (Beckman Coulter); Spectroflo (Cytek); FACSDiva v6 (Becton Dickinson) EpiCenter v4.00A-PSO(Becton Dickinson); bcf2fastq Conversion Software (Illumina)

Data analysis: GraphpadPrism v8.1&v9.3.1; Microsoft Excel (Office 365); bcf2fastq Conversion Software (Illumina) 10x Genomics STAR in CellRanger pipeline (v3.1.0) ; souporcell (v2.0) ;. Seurat (v4.0.0) package of R (v4.0.2), ClusterProfiler (v3.18.1) R- packages: NormalizeData; FindVariableFeatures, SelectIntegrationFeatures, IntegrateData, SingleR package of R HumanPrimaryCellAtlasData, BlueprintEncodeData, MonacoImmuneData, DatabaseImmuneCellExpressionData and NovershternHematopoieticData;enrichGO and enrichKEGG; Ingenuity pathway analysis (Qiagen); Morpheus software (Broad Institute); OMIQ (Dotmatics); R (v1.4.1717) package dplyr, libraries dplyr, tidyverse, tidyr, ggplot2, reshape plotrix, ggrepel, viridis

For manuscripts utilizing custom algorithms or software that are central to the research but not yet described in published literature, software must be made available to editors and reviewers. We strongly encourage code deposition in a community repository (e.g. GitHub). See the Nature Portfolio guidelines for submitting code & software for further information.

Data

Policy information about availability of data

All manuscripts must include a data availability statement. This statement should provide the following information, where applicable:

- Accession codes, unique identifiers, or web links for publicly available datasets
- A description of any restrictions on data availability
- For clinical datasets or third party data, please ensure that the statement adheres to our policy

All data generated or analyzed during this study are included in this published article (and its supplementary information files in particular the source data file).
Single cell RNA sequencing data of the current study is deposited in the European Genome-Phenome Archive repository with ID: EGAS00001006990 [https://ega-archive.org/search-results.php?query=EGAS00001006990].
Canonical pathways analysis identified the pathways from the QIAGEN Ingenuity Pathway Analysis library with the QIAGEN's Knowledge Base.
Source data are provided with this paper.

Research involving human participants, their data, or biological material

Policy information about studies with human participants or human data. See also policy information about sex, gender (identity/presentation), and sexual orientation and race, ethnicity and racism.

Reporting on sex and gender	i)Our Dutch cohort comprised 20 women (average age 24.3 years (19.7 – 54.9 yrs)) and 22 men (average age 27.5 years (18.5 – 70.6 yrs)) identified by self-reporting and were thereby sex and age matched (source data file)<br>ii) Indian validation cohort consisting of 58 individuals (age 18-24) of whom 33 were studied in more detail of which 19 are female and 14 are male identified by selfreporting (source data file)<br>results influenced by gender are discussed in the manuscript, all individual data are in the source data file
Reporting on race, ethnicity, or other socially relevant groupings	the 300BCG cohort consists of healthy adult volunteers of Western European ancestry;<br>the Indian BCG revaccination cohort included healthcare workers recruited at St.John's Medical College-Hospital in Bangalore, India
Population characteristics	i)Our Dutch cohort of western european origin has an average age of 24.3 years (19.7 – 54.9 yrs) for the women included and an average age 27.5 years (18.5 70.6 yrs) for the men (source data file)<br>ii)The Indian validation cohort consisting of 58 individuals (age 18-24) of whom 33 were studied in more detail of which 19 are female and 14 are male identified by self-reporting (source data file).
Recruitment	The Dutch cohort was recruited by voluntary registration.<br>The Indian healthcare workers of St. John's Medical College-Hospital, Bangalore, India were invited to participate in the study from October 2019 to June 2021 .Within cohort data analysis has been performed, thus independent of recruitment regimen
Ethics oversight	The Dutch cohort study was approved by the Arnhem-Nijmegen Medical Ethical Committee with number NL58553.091.16. The Indian healthcare workers of St. John's Medical College-Hospital, Bangalore, India study was approved by Institutional Ethics Review Committee of St. John Medical College Hospital, Bangalore, IEC Ref no:(IEC/1/896/2018).<br>All studies were performed in accordance with the declaration of Helsinki.

Note that full information on the approval of the study protocol must also be provided in the manuscript.

Field-specific reporting

Please select the one below that is the best fit for your research. If you are not sure, read the appropriate sections before making your selection.

☒ Life sciences ☐ Behavioural & social sciences ☐ Ecological, evolutionary & environmental sciences

For a reference copy of the document with all sections, see nature.com/documents/nr-reporting-summary-flat.pdf

Life sciences study design

All studies must disclose on these points even when the disclosure is negative.

Sample size	no sample size calculation was performed, sample size was based on previous funtional assay based studies where groups sizes of 20 participants provided significant results.<br>The 300BCG cohort selection used in this study represented just over 10% of cohort participants and were selected based on the IL-1b response against S.aureus.<br>All participants of the Indian BCG revaccination study of which one PBMC vial was available were included in this study.
Data exclusions	no data were excluded and all is provided in the source data file
Replication	The MGIA experiments were performed in duplicate in independent experimental runs. Each PBMC sample was tested once as only one vial of these unique vaccination cohort samples were availble for functional assesment.<br>Assay controls for determining the mycobacterial concentrations and calculating the regression lines were included in every experiment. Inoculum controls were performed in duplicate for both the MGIT tubes as well as the CFU plating.<br>When enough PBMCs were available samples were processed for flowcytometry and included.No available data was excluded from the analysis.<br>This study was performed with the 300BCG cohort as primary study group and validated by the Indian BCG revaccination cohort.
Randomization	samples from each cohort were tested independently and for each cohort samples were randomizated but the corrsponding pre and post vaccination samples were paired in the assays.<br>For the MGIA, 12 to 20 indviduals were randomly distibuted by the original study physician over 3 experimental runs and tested. Samples of the 300BCG cohort were blinded to the researchers while performing experiments.<br>The samples of the Indian revaccination study were also randomly distributed over 3 experimental runs with equal distribution of BCG revaccinee and non revaccinee samples and pairing of pre and post BCG-revaccination samples.
Blinding	Investigators were blinded to group allocation

Behavioural & social sciences study design

All studies must disclose on these points even when the disclosure is negative.

Study description	
Research sample	
Sampling strategy	
Data collection	
Timing	
Data exclusions	
Non-participation	
Randomization	

Ecological, evolutionary & environmental sciences study design

All studies must disclose on these points even when the disclosure is negative.

Study description	
Research sample	
Sampling strategy	
Data collection	
Timing and spatial scale	
Data exclusions	
Reproducibility	
Randomization	
Blinding	

Did the study involve field work? ☐ Yes ☐ No

Field work, collection and transport

Field conditions	
Location	
Access & import/export	
Disturbance	

Reporting for specific materials, systems and methods

We require information from authors about some types of materials, experimental systems and methods used in many studies. Here, indicate whether each material, system or method listed is relevant to your study. If you are not sure if a list item applies to your research, read the appropriate section before selecting a response.

Materials & experimental systems

n/a	Involved in the study
☐	☒ Antibodies
☒	☐ Eukaryotic cell lines
☒	☐ Palaeontology and archaeology
☒	☐ Animals and other organisms
☒	☐ Clinical data
☒	☐ Dual use research of concern
☒	☐ Plants

Methods

n/a	Involved in the study
☒	☐ ChIP-seq
☐	☒ Flow cytometry
☒	☐ MRI-based neuroimaging

Antibodies

Antibodies used	see supplementary data tables 1 and 2;
Validation	All monoclonal antibodies used for flow cytometry were commercially available and titrated to obtain optimal signal to noise ratios, comparing the specific staining to a gold standard or previously published monoclonal antibody to ensure the staining is biologically appropriate.

Eukaryotic cell lines

Policy information about cell lines and Sex and Gender in Research

Cell line source(s)	NA
Authentication	NA
Mycoplasma contamination	NA
Commonly misidentified lines (See ICLAC register)	NA

Palaeontology and Archaeology

Specimen provenance	
Specimen deposition	
Dating methods	

☐ Tick this box to confirm that the raw and calibrated dates are available in the paper or in Supplementary Information.

Ethics oversight	

Note that full information on the approval of the study protocol must also be provided in the manuscript.

Animals and other research organisms

Policy information about studies involving animals; ARRIVE guidelines recommended for reporting animal research, and Sex and Gender in Research

Laboratory animals	
Wild animals	
Reporting on sex	
Field-collected samples	
Ethics oversight	

Note that full information on the approval of the study protocol must also be provided in the manuscript.

Clinical data

Policy information about clinical studies

All manuscripts should comply with the ICMJE guidelines for publication of clinical research and a completed CONSORT checklist must be included with all submissions.

Clinical trial registration	
Study protocol	
Data collection	
Outcomes	

Dual use research of concern

Policy information about dual use research of concern

Hazards

Could the accidental, deliberate or reckless misuse of agents or technologies generated in the work, or the application of information presented in the manuscript, pose a threat to:

No	Yes	
☒	☐	Public health
☒	☐	National security
☒	☐	Crops and/or livestock
☒	☐	Ecosystems
☒	☐	Any other significant area

Experiments of concern

Does the work involve any of these experiments of concern:

No	Yes	
☒	☐	Demonstrate how to render a vaccine ineffective
☒	☐	Confer resistance to therapeutically useful antibiotics or antiviral agents
☒	☐	Enhance the virulence of a pathogen or render a nonpathogen virulent
☒	☐	Increase transmissibility of a pathogen
☒	☐	Alter the host range of a pathogen
☒	☐	Enable evasion of diagnostic/detection modalities
☒	☐	Enable the weaponization of a biological agent or toxin
☒	☐	Any other potentially harmful combination of experiments and agents

Plants

Seed stocks

Novel plant genotypes

Authentication

ChIP-seq

Data deposition

☐ Confirm that both raw and final processed data have been deposited in a public database such as GEO.

☐ Confirm that you have deposited or provided access to graph files (e.g. BED files) for the called peaks.

Data access links
May remain private before publication.

Files in database submission

Genome browser session
(e.g. UCSC)

Methodology

Replicates

Sequencing depth

Antibodies

Peak calling parameters

Data quality

Software

Flow Cytometry

Plots

Confirm that:

- [x] The axis labels state the marker and fluorochrome used (e.g. CD4-FITC).
- [x] The axis scales are clearly visible. Include numbers along axes only for bottom left plot of group (a 'group' is an analysis of identical markers).
- [x] All plots are contour plots with outliers or pseudocolor plots.
- [x] A numerical value for number of cells or percentage (with statistics) is provided.

Methodology

Sample preparation

PBMCs were thawed, rested for 2 hours in DNAse, washed and stained according to description in Methods
Freshly isolated PBMCs were stained according to description in Methods

Instrument

10-color Navios flow cytometer (Beckman Coulter) equipped with three solid-state lasers (488 nm, 638 nm, and 405 nm); a 5L Cytek®Aurora (Cytek Biosciences, Fremont, CA, USA) ;
BD Aria Fusion

Software

Flowjo v10; FACS Diva ; Kaluza; Omiq.ai

Cell population abundance

NA

Gating strategy

i)the freshly isolated PBMCs from the Dutch BCG vaccination cohort was gated on Time vs FCS, followed by a single cell gate by FSC-A vs FSC-H and a CD45+ gate by CD45 vs SSC.
All CD45+ events were selected and CD3+ T-cells were selected, CD3-CD56- cells were used as a pre-gate for the CD14 and CD16 monocytes. CD38+events against the CD24 were used to define B cell populations identified by CD19 and CD20.
ii) the fresh PBMCs from the Indian cohort were first selected on single cells by the FCS-A vs FCS-H, followed by CD3+ selection against SSC and a CD4 or CD8 T-cell gate was set.
IFNg positivity was defined against a FMO control.
iii) Frozen PBMCs form the Indian cohort were gated against time, followed by single cells through FCS-A vs FSC-H, dead cells were excluded by a live/dead marker against SSC.
Lymphogate was set by FSC-A vs SSC-A and T cells were selected By CD3 positivity against CD14. NK and NKT cells were discriminated by CD3 and CD56, B cells by CD19 and CD20 and myeloid cells were characterized into the different monocyte subsets by the CD14 and CD16 combination. For all gates examples are shown in supplementary figure 4

- [x] Tick this box to confirm that a figure exemplifying the gating strategy is provided in the Supplementary Information.

Magnetic resonance imaging

Experimental design

Design type

Design specifications

Behavioral performance measures

Imaging type(s)

Field strength

Sequence & imaging parameters

Area of acquisition

Diffusion MRI ☐ Used ☐ Not used

Preprocessing

Preprocessing software

Normalization

Normalization template

Noise and artifact removal

Volume censoring

Statistical modeling & inference

Model type and settings

Effect(s) tested

Specify type of analysis: ☐ Whole brain ☐ ROI-based ☐ Both

Statistic type for inference

(See Eklund et al. 2016)

Correction

Models & analysis

n/a	Involved in the study
☐	☐ Functional and/or effective connectivity
☐	☐ Graph analysis
☐	☐ Multivariate modeling or predictive analysis

Functional and/or effective connectivity

Graph analysis

Multivariate modeling and predictive analysis

This checklist template is licensed under a Creative Commons Attribution 4.0 International License, which permits use, sharing, adaptation, distribution and reproduction in any medium or format, as long as you give appropriate credit to the original author(s) and the source, provide a link to the Creative Commons license, and indicate if changes were made. The images or other third party material in this article are included in the article's Creative Commons license, unless indicated otherwise in a credit line to the material. If material is not included in the article's Creative Commons license and your intended use is not permitted by statutory regulation or exceeds the permitted use, you will need to obtain permission directly from the copyright holder. To view a copy of this license, visit http://creativecommons.org/licenses/by/4.0/
